# Ginkgo Flavonol Glycosides or Ginkgolides Tend to Differentially Protect Myocardial or Cerebral Ischemia–Reperfusion Injury *via* Regulation of TWEAK-Fn14 Signaling in Heart and Brain

**DOI:** 10.3389/fphar.2019.00735

**Published:** 2019-07-05

**Authors:** Guangxu Xiao, Ming Lyu, Yule Wang, Shuang He, Xinyan Liu, Jingyu Ni, Lan Li, Guanwei Fan, Jihong Han, Xiumei Gao, Xiaoying Wang, Yan Zhu

**Affiliations:** ^1^Tianjin State Key Laboratory of Modern Chinese Medicine, Tianjin University of Traditional Chinese Medicine, Tianjin, China; ^2^Research and Development Center of TCM, Tianjin International Joint Academy of Biotechnology & Medicine, Tianjin, China; ^3^Institute of Chinese Materia Medica, China Academy of Chinese Medicial Sciences, Beijing, China; ^4^Medical Experiment Center, First Teaching Hospital of Tianjin University of Traditional Chinese Medicine, Tianjin, China; ^5^College of Life Sciences, Key Laboratory of Medicinal Chemical Biology, Key Laboratory of Bioactive Materials of Ministry of Education, Nankai University, Tianjin, China; College of Biomedical Engineering, Hefei University of Technology, Hefei, China; ^6^Neuroprotection Research Laboratory, Departments of Radiology and Neurology, Massachusetts General Hospital, Harvard Medical School, Charlestown, MA, United States

**Keywords:** Shuxuening injection, ginkgo flavonol glycosides, ginkgolides, myocardial ischemia—reperfusion injury, cerebral ischemia—reperfusion injury, TWEAK–Fn14 signaling

## Abstract

Shuxuening injection (SXNI), one of the pharmaceutical preparations of *Ginkgo biloba* extract, has significant effects on both ischemic stroke and heart diseases from bench to bedside. Its major active ingredients are ginkgo ﬂavonol glycosides (GFGs) and ginkgolides (GGs). We have previously reported that SXNI as a whole protected ischemic brain and heart, but the active ingredients and their contribution to the therapeutic effects remain unclear. Therefore, we combined experimental and network analysis approach to further explore the specific effects and underlying mechanisms of GFGs and GGs of SXNI on ischemia–reperfusion injury in mouse brain and heart. In the myocardial ischemia–reperfusion injury (MIRI) model, pretreatment with GFGs at 2.5 ml/kg was superior to the same dose of GGs in improving cardiac function and coronary blood flow and reducing the levels of lactate dehydrogenase and aspartate aminotransferase in serum, with an effect similar to that achieved by SXNI. In contrast, pretreatment with GGs at 2.5 ml/kg reduced cerebral infarction area and cerebral edema similarly to that of SXNI but more significantly compared with GFGs in cerebral ischemia–reperfusion injury (CIRI) model. Network pharmacology analysis of GFGs and GGs revealed that tumor necrosis factor-related weak inducer of apoptosis (TWEAK)–fibroblast growth factor-inducible 14 (Fn14) signaling pathway as an important common mechanism but with differential targets in MIRI and CIRI. In addition, immunohistochemistry and enzyme linked immunosorbent assay (ELISA) assays were performed to evaluate the regulatory roles of GFGs and GGs on the common TWEAK–Fn14 signaling pathway to protect the heart and brain. Experimental results confirmed that TWEAK ligand and Fn14 receptor were downregulated by GFGs to mitigate MIRI in the heart while upregulated by GGs to improve CIRI in the brain. In conclusion, our study showed that GFGs and GGs of SXNI tend to differentially protect brain and heart from ischemia–reperfusion injuries at least in part by regulating a common TWEAK–Fn14 signaling pathway.

## Introduction

Cardio-cerebral vascular diseases, including mainly ischemic myocardial infarction and ischemic stroke, are the most common causes of disability and death worldwide (Benjamin et al., 2018). It is well-established that ischemia–reperfusion (I/R) injury of both heart and brain shares certain common pathological mechanisms such as inflammation (Iadecola and Anrather, 2011; Goldfine and Shoelson, 2017), oxidative stress (Sanderson et al., 2013; Muntean et al., 2016), microvascular dysfunction (Gursoy-Ozdemir et al., 2012; Granger and Kvietys, 2017), and mitochondrial dysfunction (Ago et al., 2010; Yang et al., 2015). Recently, increasing numbers of research have explored underlying mechanisms of the intimate internal connection between ischemic stroke and heart diseases (Prosser et al., 2007; Samuels, 2007; Ishikawa et al., 2013; Abete et al., 2014; Gladstone et al., 2014; Sanna et al., 2014; Chen et al., 2017c; Lyu et al., 2017; Lyu et al., 2018). In recent years, a growing number of studies indicated that certain traditional Chinese medicines (TCMs) and their main active ingredients have protective and curative effects on I/R-mediated injury in brain and heart (Han et al., 2017).

Extracts of *Ginkgo biloba* (EGB) is one of the most common and earliest industrialized herbal medicinal preparations. As a pharmaceutical brand of EGB, Shuxuening injection (SXNI) is approved by the Sino Food and Drug Administration, and its major identified active ingredients consist of consist of ginkgo flavonol glycosides (GFGs) and ginkgolides (GGs). EGB has a broad range of pharmacological effects, such as anti-inﬂammation (Chen et al., 2017b), antioxidant (Saini et al., 2014), anti-apoptotic (Yu et al., 2018), antidepressant (Hu et al., 2018), anti-arrhythmia (Zhao et al., 2013), antitumor (Li et al., 2017), improving cognitive function (Wan et al., 2016), and relieving I/R injury (Jin et al., 2014; Ran et al., 2014). Most recently, its therapeutic effect on cardio-cerebrovascular diseases has attracted renewed attention (Yang et al., 2017). Meanwhile, research on GFGs and GGs is deepening. So far, identified GFGs include quercetin, kaempferol, myricetin, apigenin, isorhamnetin, luteolin, and tamarixetin, whereas identified GGs consist of ginkgolide A, ginkgolide B, ginkgolide C, ginkgolide J, ginkgolide M, ginkgolide K, ginkgolide L, ginkgolide P, ginkgolide Q, and bilobalide (van Beek and Montoro, 2009; Liao et al., 2011; Mohanta et al., 2014). Taking quercetin in GFGs as an example, its variety of pharmacological activities include anti-inflammatory (Song et al., 2018), anti-oxidation (Fadda et al., 2018), anticancer (Patra et al., 2018), anti-infective (Mehrbod et al., 2018), hypotensive (Marunaka et al., 2017), and hypoglycemic (Zhuang et al., 2018). The role of quercetin in the treatment of cardiovascular diseases and neurodegenerative disease is also frequently reported (Enogieru et al., 2018; Patel et al., 2018). The pharmacological effects of GGs are also extensive, mainly including anti-apoptotic (Guo et al., 2015), neuroprotection (Zheng et al., 2016), antioxidative stress (Priyanka et al., 2014), anti-arrhythmia (Zhao et al., 2013), and antiplatelet (Rui et al., 2016). Consequently, more attention has been drawn to studying the underlying active ingredients and molecular mechanisms of SXNI in preventing and treating ischemia stroke and coronary heart disease.

Tumor necrosis factor (TNF)-related weak inducer of apoptosis (TWEAK), also named as TNFSF12, APO3L, or CD255, is a member of the TNF superfamily (Chicheportiche et al., 1997; Marsters et al., 1998), while fibroblast growth factor-inducible 14 (Fn14), also named as TNFRSF12A, TWEAKR, or CD266 (Meighan-Mantha et al., 1999; Wiley et al., 2001), is the only known TWEAK receptor (Bossen et al., 2006). TWEAK–Fn14 axis has shown an increasingly important role in cardio-cerebral vascular diseases (Blanco-Colio, 2014). The interaction of TWEAK and Fn14 activates downstream signaling processes during disease development and progression, which includes mediating atrial-derived HL-1 myocytes hypertrophy *via* the JAK2/STAT3 signaling pathway (Hao et al., 2018), weakening the antiproliferative effects of miR-149 in osteosarcoma *via* the AKT serine/threonine kinase (PI3K/AKT) signaling pathway (Xu et al., 2018), inducing pro-fibrotic responses leading to heart failure *via* the NF-κB and/or AP-1 signaling pathway (Das et al., 2018), preventing renal damage in patients with lupus nephritis *via* type I interferon signaling pathway (Xue et al., 2017), and promoting wound healing processes through favoring regional inﬂammation, cytokine production, and extracellular matrix synthesis (Liu et al., 2018). In addition, TWEAK also participates in a variety of other diseases such as tumors (Hu et al., 2017), neonatal hypoxia–ischemia (Kichev et al., 2018), human glioma (Guan et al., 2017), psoriasis (Sidler et al., 2017), and chronic colitis (Son et al., 2013).

We have previously reported that SXNI reduces both myocardial and cerebral I/R injuries in heart and brain *via* a common protection mechanism involving Tnfrsf12a-mediated atherosclerosis signaling and inflammatory response (Lyu et al., 2018). However, since SXNI contains multicomponents, the specific contribution by different ingredients and their pharmacological mechanisms remain poorly understood in this setting. In the present study, we deciphered the roles of GFGs and GGs and confirmed that GFGs were superior than GGs in exhibiting the protective effect on myocardial I/R injury *via* suppressing TWEAK–Fn14 signaling and GGs protected cerebral I/R injury more effectively compared with GFGs by promoting TWEAK–Fn14 signaling. Our new ﬁndings may contribute to further understanding of the organ-specific therapeutic effects of SXNI and provide a new basis for the clinical application of EGBs, which will also promote the development and application in TCM.

## Materials and Methods

### Drugs and Reagents

SXNI (drug approval number: Z13020795; batch number: 15101201), GFGs, and GGs were supplied by China Shineway Pharmaceutical Group Ltd. (Shijiazhuang, China). According to manufacturer’s instruction, the chemical content of one SXNI dose (5 ml/unit) is equal to 17.5 mg *Ginkgo biloba* leaf extract, which includes 4.2 mg total GFGs and 0.7 mg GGs. GFGs (0.84 mg/ml) and GGs (0.14 mg/ml) are also supplied as injections (Ma et al., 2018). We bought 2, 2, 2-Tribromoalcohol from Sigma (T48402, St. Louis, MO, United States). High-efficiency radioimmunoprecipitation assay tissue/cell lysate (R0010), 2, 3, 5-Triphenyl-2H-tetrazolium chloride (TTC, T8170), and bicinchoninic acid (PC0020) protein concentration assay kit were purchased from Solarbio (Beijing, China). Anti-TWEAKR antibody was purchased from Abcam (EPR3179, Shanghai, China). Mouse ELISA kits for TWEAK, interleukin 6, and annexin A1 were purchased from ZCi BiO (Shanghai, China). Other ELISA kits for lactate dehydrogenase (LDH), aspartate aminotransferase (AST), creatine kinase-MB (CK-MB), and creatine kinase (CK) were purchased from Biosino Bio-Technology and Science Inc. (Beijing, China).

### Animals

Male 6-week-old C57BL/6J mice (22 ± 2 g) were purchased from Beijing Vital River Laboratory Animal Technology Co., Ltd. (Beijing, China, Certiﬁcate no.: SCXK Jing 2016-0006). This study was carried out in accordance with the principles of the Basel Declaration and recommendations of the Care and Use of Laboratory Animals issued by the Ministry of Science and Technology of China. The protocol was approved by the Laboratory Animal Ethics Committee of Tianjin University of TCM (Permit Number: TCM-LAEC2014004). The experimental animals were previously randomized into several groups and kept with commercial rat food and water. They were housed under controlled temperature (22°C) and relative humidity (40% ± 5%) conditions with a 12-h light/dark cycle.

### Establishment of Myocardial Ischemia–Reperfusion Injury Model and Drug Administration

A mouse model of myocardial I/R injury (MIRI) was performed as previously described (Gao et al., 2010). Briefly, mice were anesthetized by inhalation of 3% isoflurane driven by 100% O_2_ in an induction chamber and then moved to an operating table in a supine position and kept under anesthesia using an inhalation mask and 2% isoflurane (100 ml/min O_2_). Next, the chest of mouse was opened at the intercostal space between 3 and 4 sternal rib *via* a left thoracotomy. Once the heart was squeezed out, the proximal left anterior descending coronary artery under left auricle is transiently ligated by a slipknot utilizing a 7-0 silk suture. Following the thorax closure and as soon as spontaneous respiration was sufficient, the mice were released and placed on an electric blanket. After 30 min of ischemia, the slipknot was untied and followed by 24 h of reperfusion. ST-segment elevation on an electrocardiogram monitor represented a success in MIRI model surgery. In sham control mice, the surgical procedures were identical, except the left anterior descending coronary artery was not tied. The mice were stochastically divided into ﬁve groups in the following experiments: sham, I/R (saline), I/R+SXNI (dose, 2.5 ml/kg), I/R+GFGs (dose, 2.5 ml/kg), and I/R+GGs (dose, 2.5 ml/kg). SXNI, GFGs, GGs, and saline were administered *via* intravenous tail injection within 10 min after the onset of ischemia. Sham and I/R groups were intravenously administered with 0.9% normal saline. No differences in operative mortality were observed among groups investigated.

### Establishment of Cerebral Ischemia–Reperfusion Injury Model and Drug Administration

For preparation of cerebral I/R injury (CIRI) model (Rousselet et al., 2012), anesthesia was induced with 2% isoﬂurane inhalation and then maintained with 1.5% isoﬂurane using a small animal respirator (RWD, Inc., China). After a midline incision at the neck, the right common carotid artery, internal carotid artery (ICA), and external carotid artery (ECA) were exposed *via* careful blunt separation, and the occipital, the cranial thyroid, the pterygopalatine artery, and the ascending pharyngeal artery were cauterized utilizing a monopolar electrical cautery. After occlusion of the right common carotid artery and ICA using two micro-clips, the right distal ECA was tightly tied by two straps using a sterile 6-0 silk suture and interrupted from the middle. Then, a small hole was made in the right ECA utilizing a micro-scissor, and a silicone-coated 4-0 nylon monoﬁlament (Jialing Biotechnology Co., Ltd., Guangzhou, China) was introduced into the right ECA and gently advanced through the right ICA until its tip blocked the origin of the right middle cerebral artery (MCA), leading a decline of local cortical blood ﬂow in the right MCA territory to 20–30% of baseline. After 60 min of ischemia, the blood flow was restored by allowing the ﬁlament withdrawal and followed by 24 h of reperfusion. Sham-operated mice were operated identically except for the MCA occlusion procedure. The mice were then randomly divided into five groups: sham, I/R (saline), I/R+SXNI (dose, 2.5 ml/kg), I/R+GFGs (dose, 2.5 ml/kg), and I/R+GGs (dose, 2.5 ml/kg). Drug and saline were injected through the vein after 50 min of ischemia.

### Echocardiographic Measurement

The noninvasive assessment of left ventricular function and coronary blood flow in the heart were made using an ultra-high resolution small animal ultrasound imaging system (Vevo 2100, VisualSonics, Toronto, ON, Canada) equipped with a 30-MHz transducer (Chen et al., 2016; Li et al., 2016). Mice were anesthetized by inhaling 1.5–2.0% isoflurane and transferred to the dorsal position and placed on a heated imaging platform. Parameters indicating cardiac functions were measured by M-mode and color Doppler mode as follows: left ventricular (LV) ejection fraction (EF), LV fractional shortening (FS), cardiac output (CO), stroke volume, LV internal dimensions at diastole (LVIDd), LV internal diameter systole (LVIDs), LV posterior wall diastole (LVPWd), LV posterior wall systole (LVPWs), LV systole volume (LV Vols), LV diastole volume (LV Vold), heart rate and LV mass, along with aortic valve (AV) peak velocity, AV peak pressure, and aorta velocity–time integral mean velocity (AoV VTI).

### Hemodynamic Evaluation of Left Ventricular Function

After 24 h of reperfusion, the cannulation connecting the biofunction experiment system (MP100-CE, BIOPAC Systems, Inc., United States) was inserted into the left ventricle through right carotid artery (Chen et al., 2015; Wang et al., 2017). Left ventricular maximum upstroke velocity (+dp/dt_max_) and left ventricular maximum descent velocity (−dp/dt_max_) were measured to reflect and evaluate the left ventricular systolic and diastolic function.

### Measurement of Biochemical Parameters

At 24 h after myocardial ischemia and reperfusion, blood was collected, and serum was separated by centrifugation at 3,000 rpm for 15 min. Concentrations of LDH, AST, CK-MB, and CK in mouse plasma were measured by ELISA kits in automatic biochemical analyzer (Multiskan MK3; Thermo Fisher Scientiﬁc, Waltham, MA, United States) according to the manufacturer’s instructions. These biochemical parameters, commonly used in clinical diagnostics to detect myocardial inﬂammation and acute myocardial infarction, were used to evaluate the myocardial damage in this research.

### Hematoxylin and Eosin Staining

After collecting the blood samples, the myocardial tissues were rapidly excised, rinsed in 0.9% saline, and ﬁxed in 10% paraformaldehyde solution for more than 48 h. Heart tissues were then cut into four 5-mm-thick slices perpendicular to the long axis of the heart and dehydrated in the automatic dehydrator (Excelsior, Thermo., Ltd., United States) with a following program: 70% alcohol for 1 h, 80% alcohol for 1 h, 90% alcohol for 1 h, 95% alcohol for 1 h, anhydrous alcohol I for 1 h, anhydrous alcohol II for 1 h, xylene I for 40 min, xylene II for 40 min, xylene III for 40 min, paraffin I for 2 h, paraffin for II 2 h, and paraffin III for 2 h. At the end of the procedure, the tissues were removed and embedded in paraffin. The paraffin blocks were pruned and cut into serial slices of 5-mm thick using a manual slicer (HM355S, Thermo., Ltd., United States), which were then placed into the automatic dyeing machine (Gemini, Thermo., Ltd., United States) for staining. The following staining routine was compiled: tissue dewaxing liquid I for 5 min, tissue dewaxing liquid II for 5 min, tissue dewaxing liquid III for 5 min, anhydrous alcohol for 5 min, anhydrous alcohol for 3 min, 95% alcohol for 1 min, 85% alcohol for 1 min, 75% alcohol for 1 min, acid alcohol for 5s, running water wash for 1 min, hematoxylin for 7 min, running water wash for 1 min, acid alcohol for 5 s, running water wash for 1 min, bluing reagent for 30 s, running water wash for 1 min, aqueous eosin for 3 min, running water wash for 1 min, 75% alcohol for 30 s, 85% alcohol for 30 s, 95% alcohol for 30 s, 100% alcohol for 1 min, 100% alcohol for 1 min, xylene I for 1 min, xylene II for 1 min, and xylene III for 1 min. After the sections were mounted by using automatic sealing machine (ClearVue, Thermo., Ltd., United States), myocardial tissues of pathological changes were observed by optical microscope (DP71, OLYMPUS, Japan).

### Quantitative Measurement of Cerebral Infarct Size

At 24 h after reperfusion, brains of euthanized mice were removed. The cerebral tissues were crosscut into five pieces of 2-mm-thick slices in a brain matrix device, which were added to the 2% solution of TTC in 0.1 M phosphate buffered solution (PBS), incubated at 37°C in dark for 10 min. After staining by TTC, the sections were photographed and used to measure the cerebral infarct size using ImageJ analysis software (National Institutes of Health, Bethesda, MD, United States) and calculated as the proportion of cerebral infarct size (percentage) = infarct size (while pale area)/total area of transverse slice × 100%.

### Analysis of Edema Volume

Anesthetized mice were injected with arterial angiography agent (Iohexol, 15 ml/kg) through the tail vein. Next, the mouse brain was imaged with a µCT small animal imager (Quantum FX; PerkinElmer, United States) with the following imaging parameters: voltage for 90 kV, current for 180 µA, field of view for 20 mm, scan technique for std 4.5 min, 360 views. The severity of cerebral edema was evaluated using ImageJ analysis software (National Institutes of Health, Bethesda, MD, United States) to calculate the offset distance of midline in the coronal image of the mouse brain at Bregma 0.3 mm (Park et al., 2014). The greater the offset distance of the midline, the more serious the cerebral edema.

### Neurological Deficit Score

At 24 h after reperfusion, according to the Bederson scale (Bederson et al., 1986) to assess the neurological deficit of mice, the neurological deficit score table is as follows: 0, walking normally, no observable defects; 1, forelimb flexion; 2, decreased resistance to lateral push without circling; 3, circling behavior in addition to the former symptoms; 4, ambulation difficulty or inability.

### Enzyme-Linked Immunosorbent Assay

After collecting the plasma of MIRI and CIRI mice, the hearts and brains were removed separately. The plasma was centrifuged at 1,000 r/min for 15 min to separate the serum. Fresh tissues of heart and brain were smashed using ultrasonic cell pulverizer (SCIENTZ-II D, Xinzhi Biotechnology Co., Ltd., China) and added the high-efficiency radioimmunoprecipitation assay tissue/cell lysate to collect the supernatant of tissue homogenates. TWEAK protein concentration in supernatant was determined using an ELISA kit (ZC-54137, ZCi Biotechnology Co., Ltd). according to the manufacturer’s instructions.

### Immunohistochemistry

The paraffin sections of heart and brain tissues (5-μm thick) were deparaffinized, incubated in distilled water, rinsed (3 × 5 min) in PBS-T (0.01 M PBS pH 7.4: KH_2_PO_4_ 0.02%, N_2_HPO_4_ 0.29%, KCl 0.02%, 0.8% NaCl, 0.05% bovine serum albumin (BSA), Tween-20 0.05%, 0.0015% TritonX-100), and then blocked endogenous peroxidase *via* incubating in 3% peroxide–methanol at room temperature for 10 min. After incubation for 1 h in blocking buffer (10% bovine serum albumin), the sections were stained for 2 h at 37°C using anti-TWEAKR antibody diluted with blocking buffer (1:50). Following wash in PBS-T (3 × 5 min), the sections were incubated sequentially for 1 h with each of the corresponding secondary antibody (Biotinylated Goat anti-Rabbit IgG) and the third antibody (Horseradish Peroxidase Streptavidin) at 37°C. The immunoreactivity of TWEAKR protein was visualized using 3, 3’-diaminobenzidine tetrahydrochloride hydrate. Finally, counterstaining was performed with hematoxylin, dehydrated, and mounted. The expression of TWEAKR (Fn14) was detected by optical microscope (DP71, OLYMPUS, Japan). The expression of Fn14 is quantified by calculating its integrated optical density value using ImagePro Plus software (National Institutes of Health, Bethesda, MD, United States) (Pei et al., 2013).

### Network Pharmacology Analysis

In previous studies, we have identified the signiﬁcantly differentially expressed genes after SXNI treatment in mice of MIRI and CIRI. Database of GFGs and GGs were established *via* literature mining. In addition, the corresponding targets of the compounds were obtained from the integration of Ingenuity^®^ Pathway Analysis (IPA), Traditional Chinese Medicine Systems Pharmacology Database and Analysis Platform, Traditional Chinese Medicine Integrative Database for Herb Molecular Mechanism Analysis, and other databases. Then, the above data were submitted to Ingenuity^®^ Pathway Analysis (IPA) to execute “Build-Path Explorer” module to determine the crucially target genes of GFGs and GGs.

### Statistical Analysis

Experimental data were presented as the mean ± SEM or mean ± SD. The GraphPad Prism 7 software (GraphPad Software, Inc., La Jolla, CA, United States) was utilized for statistical analysis, which was performed with Student’s two-tailed *t*-test for comparison between two groups or one-way ANOVA for multiple groups. A value of *P* < 0.05 was deﬁned as statistically signiﬁcant.

## Results

### Ginkgo Flavonol Glycosides Are Superior Than Ginkgolides in Improving Cardiac Function in Myocardial Ischemia–Reperfusion Injury Mice

**Figure 1:**
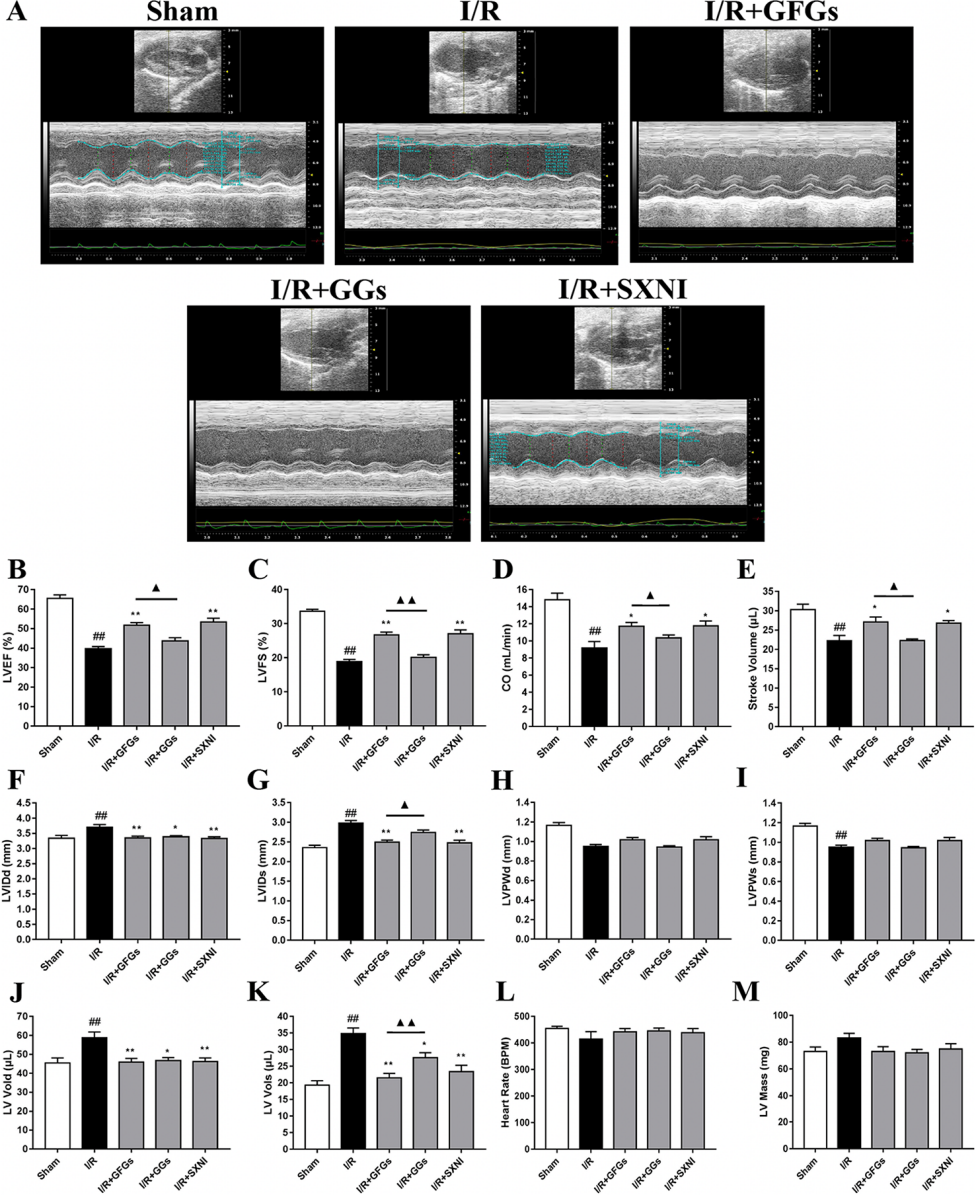
Changes of echocardiographic characterization of cardiac function in myocardial ischemia–reperfusion injury (MIRI) mice. After 30 min of ischemia and 18 h of reperfusion, the effect of ginkgo ﬂavonol glycosides (GFGs, 2.5 ml/kg), ginkgolides (GGs, 2.5 ml/kg), and Shuxuening injection (SXNI, 2.5 ml/kg) on cardiac function was quantitatively ▲ evaluated. **(A)** Representative echocardiography images of different groups. Bar graph quantitation of echocardiographic changes in cardiac function in different groups detected in M-mode: **(B)** LVEF %, **(C)** LVFS %, **(D)** CO, **(E)** stroke volume, **(F)** LVIDd, **(G)** LVIDs, **(H)** LVPWd, **(I)** LVPWs, **(J)** LV Vold, **(K)** LV Vols, **(L)** heart rate, and **(M)** LV mass. Values were expressed as mean ± SEM (*n* = 5). ^##^ *P* < 0.01 vs. sham group, *P < 0.05, **P < 0.01 vs. I/R group, ^▲^ *P* < 0.05, ^▲▲^ *P* < 0.01 vs. GFGs group (LV, left ventricular; EF, ejection fraction; FS, LV fractional shortening; CO, cardiac output; LVIDd, LV internal dimensions at diastole; LVIDs, LV internal diameter systole; LVPWd, LV posterior wall diastole; LVPWs, LV posterior wall systole; LV Vols, LV systole volume; LV Vold, LV diastole volume).

**Figure 2:**
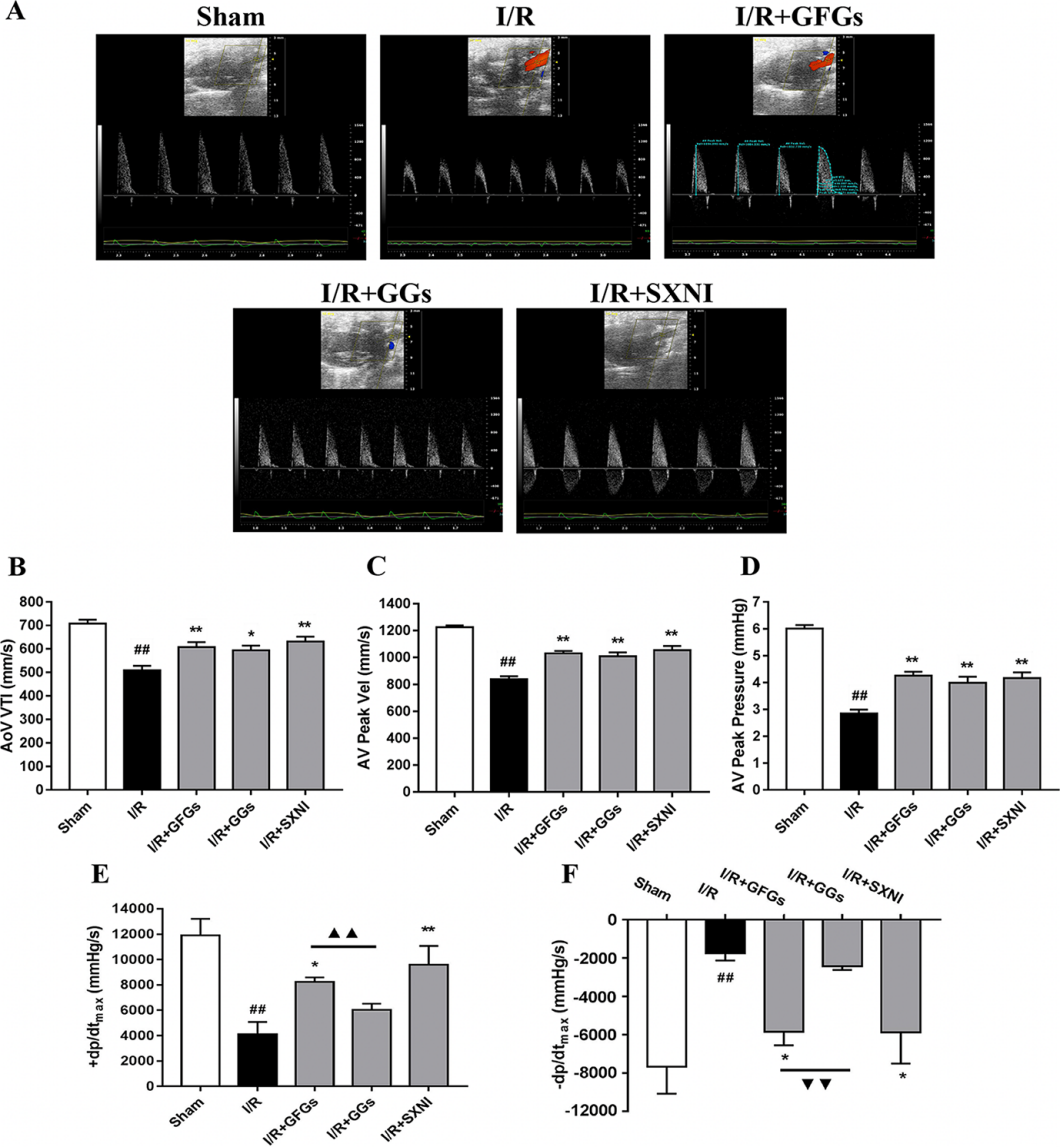
GFGs and GGs improved the coronary blood ﬂow and left ventricular function after myocardial ischemia and reperfusion in MIRI mice. After 30 min of ischemia and 18 h of reperfusion, the effect of GFGs (2.5 ml/kg), GGs (2.5 ml/kg), and SXNI (2.5 mg/kg) on cardiac function was quantitatively evaluated. **(A)** Representative echocardiography images of coronary blood ﬂow were determined with different groups. **(B)** AoV VTI, **(C)** AV peak velocity, and **(D)** AV peak pressure were detected in color Doppler mode. Quantitative assessment of hemodynamic on left ventricular function based on **(E)** +dp/dt _max_ and **(F)** −dp/dt _max_. Values were expressed as mean ± SEM (*n* = 5). ^##^ *P* < 0.01 vs. sham group, *P < 0.05, **P < 0.01 vs. I/R group, ^▲▲^ *P* < 0.01 vs. GFGs group (AV Peak Vel, aortic valve peak velocity; AoV VTI, aorta velocity–time integral mean velocity; +dp/dt_max_, left ventricular maximum upstroke velocity; −dp/dt_max_, left ventricular maximum descent velocity).

The effects of GFGs and GGs on cardiac function were first examined *via* performing the echocardiography. The I/R group signiﬁcantly decreased LVEF %, LVFS %, CO, stroke volume, and LVPWs, while the sham group had increased LVIDd, LVIDs, LV Vold, and LV Vols. No significant change was observed for LVPWd, heart rate, and LV mass (**Figure 1**). However, the marked increase in LVEF % (**Figure 1B**), LVFS % (**Figure 1C**), CO (**Figure 1D**), and stroke volume (**Figure 1E**) and decrease in LVIDd (**Figure 1F**), LVIDs (**Figure 1B**), LV Vold (**Figure 1J**), and LV Vols (**Figure 1K**) were significantly reversed by SXNI and GFGs treatment. By contrast, only decreased LVIDd (**Figure 1F**), LV Vold (**Figure 1J**), and LV Vols were observed in the I/R+GGs group (**Figure 1K**). GFGs, GGs, or SXNI had no effect on LVPWd (**Figure 1H**), LVPWs (**Figure 1I**), heart rate (**Figure 1L**), and LV mass (**Figure 1M**) compared with the I/R group. In addition, the effects of GFGs and GGs on coronary blood ﬂow were obtained in color Doppler mode at 18 h after myocardial I/R (**Figure 2A**). As the picture shows, the decline in AoV VTI (**Figure 2B**), AV peak velocity (**Figure 2C**), AV peak pressure (**Figure 2D**), left ventricular maximum upstroke velocity (+dp/dt_max_) (**Figure 2E**), and left ventricular maximum descent velocity (−dp/dt_max_) (**Figure 2F**) in the I/R group were outstanding compared with the sham group. However, GFG and SXNI treatment significantly reversed this decline compared with GGs (**Figure 2E and F**). Taken together, these data indicate that the therapeutic effect of GFGs is superior to GGs in improving cardiac function in MIRI mice.

### Ginkgo Flavonol Glycosides Are Superior to Ginkgolides in Attenuating Myocardial Injury in Myocardial Ischemia–Reperfusion Injury Mice

**Figure 3:**
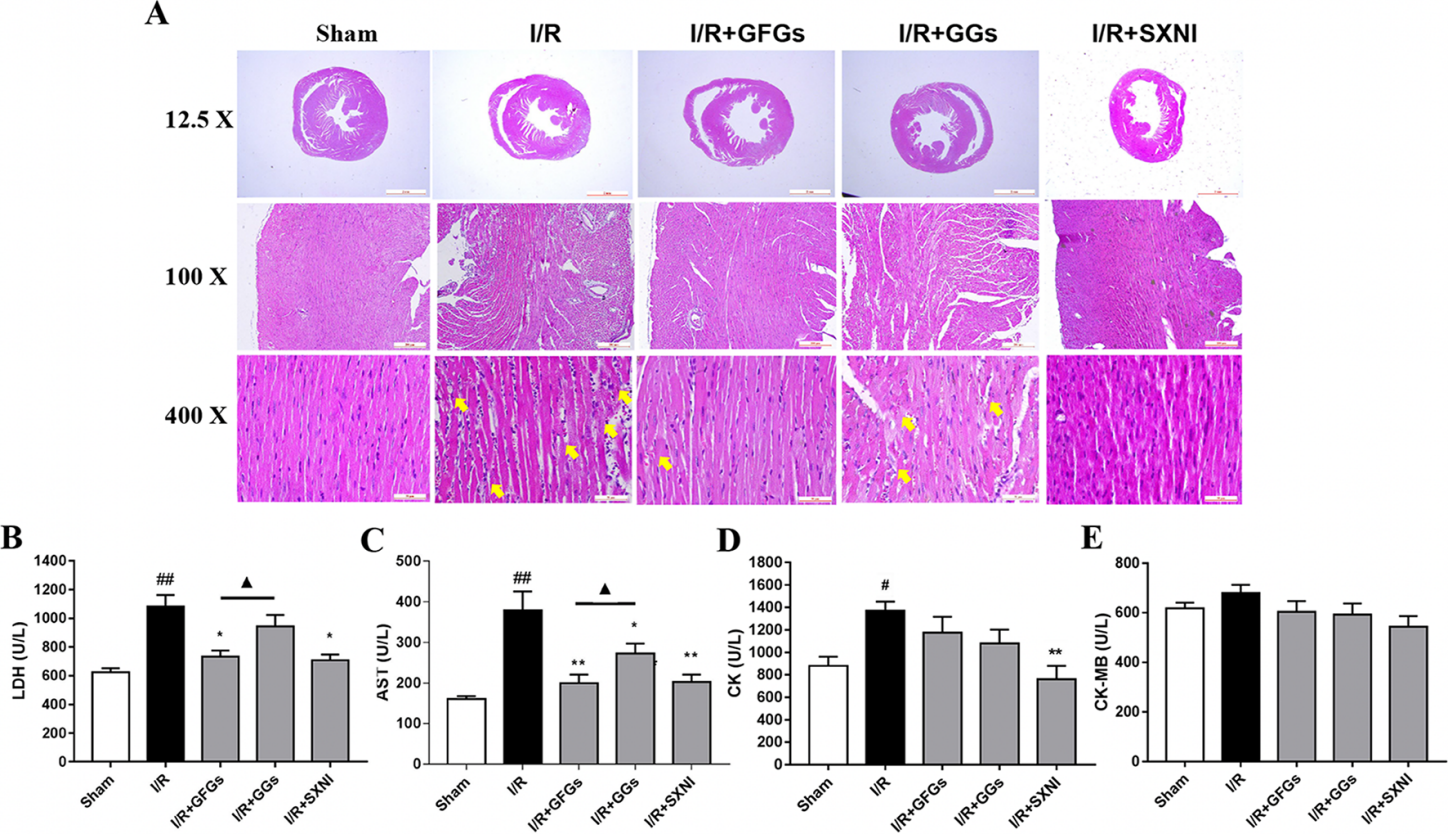
GFGs and GGs alleviated the myocardial I/R injury in MIRI mice. After 30 min of ischemia and 24 h of reperfusion, the protective effects of GFGs and GGs on MIRI were assessed by hematoxylin and eosin staining and measurement of biochemical parameters. **(A)** Hematoxylin and eosin staining results for the histopathological of the myocardial tissue indicated the histopathological changes in I/R model as well as that caused by GFGs, GGs, and SXNI. Myocardium damage sections were observed and indicated with yellow arrows in representative pictures (12.5×, 100×, and 400× magniﬁcation). **(B–E)** Effects of GFGs and GGs on the alterations of lactate dehydrogenase (LDH), aspartate aminotransferase (AST), creatine kinase-MB (CK-MB), and creatine kinase (CK) in serum following reperfusion (*n* = 6). Values were expressed as mean ± SEM. ^#^ *P* < 0.05, ^##^ *P* < 0.01 vs. sham group, *P < 0.05, **P < 0.01 vs. I/R group, ^▲^ *P* < 0.05 vs. GFGs group.

After 30-min ischemia and 24-h reperfusion, the protective effects of GFGs and GGs on myocardial injury in MIRI mice were investigated *via* observing the histopathological features of the myocardial tissue and detecting the levels of LDH, AST, CK-MB, and CK in serum. The results indicated that the elevated levels of LDH and AST caused by I/R injury were significantly reduced by GFGs and SXNI administration (**Figures 3B and C**). Furthermore, SXNI also obviously decreased the level of CK in serum (**Figure 3D**). On the other hand, GGs only marginally altered serum AST compared with the I/R group (**Figure 3C**). The results of the hematoxylin and eosin staining showed that I/R injury led to extensive myocardial structural disarray, increased necrosis and fusion area, as well as infiltration of inﬂammatory cells into myocardial tissue, which were indicated with yellow arrows (**Figure 3A**). However, GFGs and SXNI were significantly more effective than GGs in improving the histological features of damaged tissue (**Figure 3A**). Therefore, GFGs was more effective than GGs to attenuate myocardial damage in MIRI Mice.

### Ginkgolides Are Superior to Ginkgo Flavonol Glycosides in Reducing Cerebral Ischemia–Reperfusion Injury in Cerebral Ischemia–Reperfusion Injury Mice

**Figure 4:**
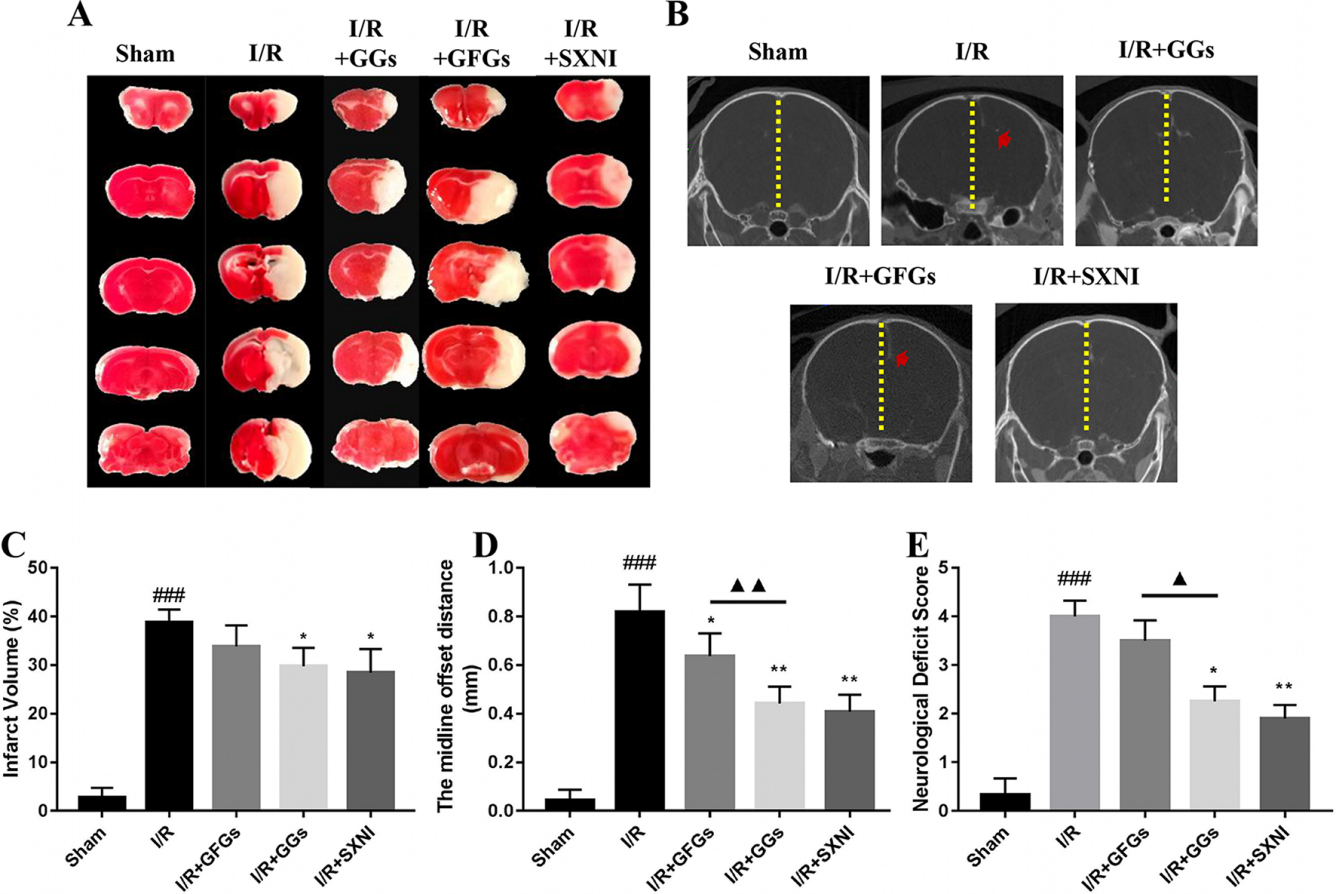
GGs reduced the cerebral infarct size, edema, and neurological deficit score in cerebral ischemia–reperfusion injury (CIRI) mice. After 60 min of ischemia and 24 h of reperfusion, brain tissue was imaged and transected for detection of cerebral damage by micro-CT and 2, 3, 5-Triphenyl-2H-tetrazolium chloride (TTC) staining, separately. Normal brain tissues were represented in red, whereas the white marked areas of infarct. **(A)** Representative images of TTC staining in different groups, including sham, I/R model, I/R+GGs, I/R+GFGs, and I/R+SNXI groups. TTC staining was performed at 24 h after stroke. **(C)** Quantitation of TTC stain of brain slices as the percentage of infarct volumes of each group (*n* = 4–5). The occurrence of cerebral edema causes the midline of the coronal image of the brain to shift (the red arrow in Figure B was the offset midline). Therefore, the more the midline offset distance, the more severe the brain edema. **(B)** Representative images of micro-CT imaging in different groups, including sham, I/R model, I/R+GGs, I/R+GFGs, and I/R+SNXI groups. **(D)** Quantitation of midline offset distance of each group (*n* = 4–5). In addition, the mice were scored for neurological deficits. **(E)** Neurological deficit score. Values were expressed as mean ± SEM. ^###^ *P* < 0.001 vs. sham group, *P < 0.05, **P < 0.05 vs. I/R group, ^▲^ *P* < 0.05, ^▲▲^ *P* < 0.01 vs. GFGs group.

The I/R injury in middle cerebral artery occlusion (MCAO) mice was estimated *via* quantifying infarct volumes in TTC- stained brain slices, indirectly calculating the severity of brain edema in micro-CT imaging and neurologic deficit scale. After 24 h of reperfusion, the cerebral infarct size, the midline offset distance, and neurologic deficit scale in I/R group were significantly increased compared with that of the sham group (**Figure 4**). GGs and SXNI treatment significantly reduced cerebral infarct size (29.7 and 28.4%, *p* < 0.05), whereas GFGs treatment showed a similar trend but did not reach statistical significance (**Figure 4A and C**). Meanwhile, although the shortening of the midline offset distance in GFGs group, which also indirectly proves that the degree of cerebral edema was reduced, was evident in the GGs, and SXNI groups, the effects of GGs and SXNI are stronger than those of GFGs (**Figure 4B and D**). Then, for the protection of neurological function, the therapeutic effects of the GGs and SXNI groups were also stronger than those of the GFGs group (**Figure 4E**). These results indicated that cerebral protection by SXNI in MCA occlusion mice is attributable mainly to GGs instead of GFGs.

### Effects of Ginkgo Flavonol Glycosides and Ginkgolides on the Expression of TWEAK and Fn14 in Myocardial Ischemia–Reperfusion Injury and Cerebral Ischemia–Reperfusion Injury Mice

**Figure 5:**
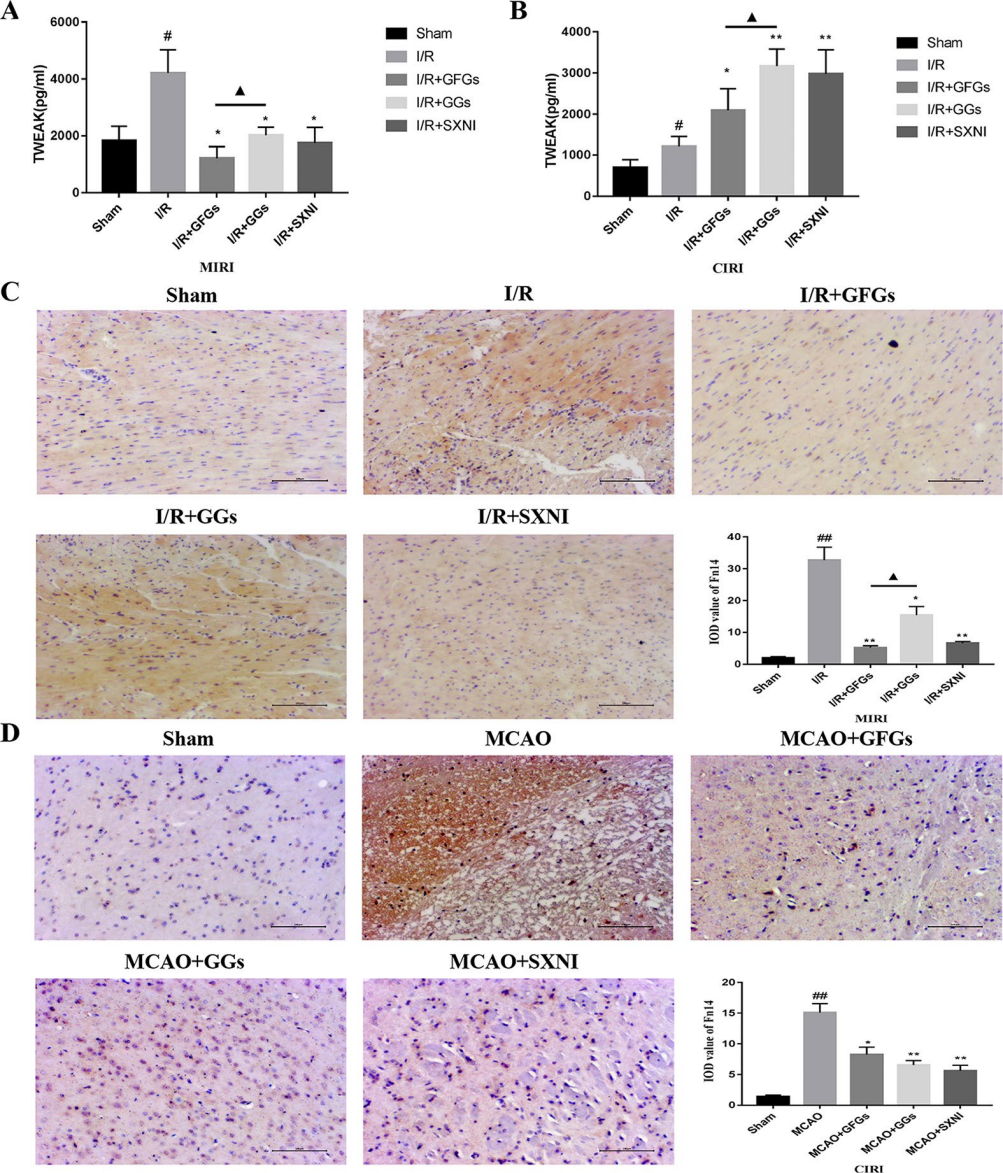
Expression levels of tumor necrosis factor-related weak inducer of apoptosis (TWEAK) and Fn14 in MIRI and CIRI mice. After 24 h of reperfusion, heart and brain were removed from the mice, and their supernatant and tissue block were prepared for immunohistochemical and ELISA analyses of the soluble TWEAK ligand and *in situ* Fn14 receptor protein, respectively. **(A)** Effects of GFGs, GGs, and SXNI on the alteration of TWEAK in heart tissue supernatant (*n* = 3). **(B)** Effects of GFGs, GGs, and SXNI on the alteration of TWEAK in brain tissue supernatant (*n* = 3). **(C)** Immunohistochemical staining for the heart tissue section of MIRI and control mice indicating the effects of GFGs, GGs, and SXNI on the expression of Fn14 protein (in brown). Representative images (100× magniﬁcation) and quantification (*n* = 3) are shown. **(D)** Immunohistochemical staining for the brain tissue section of CIRI and control mice indicating the effects of GFGs, GGs, and SXNI on the expression of Fn14 protein (in brown). Representative images (100× magniﬁcation) and quantification (*n* = 3) are shown. Values were expressed as mean ± SD B. ^##^ *P* < 0.01 vs. sham group, *P < 0.05, **P < 0.01, vs. model group, ^▲^ *P* < 0.05 vs. GFGs group.

The expression levels of TWEAK in heart and brain tissue supernatants of MIRI and CIRI mice were determined by ELISA assay. As shown in **Figure 5A and B**, TWEAK expressions in model groups (I/R group) were significantly elevated compared with the sham group in MIRI and CIRI mice. Treatment with GFGs, GGs, and SXNI obviously lowered the TWEAK expression in MIRI mice compared with the I/R group, in which GFGs is superior to GGs (**Figure 5A**). In CIRI mice, GFGs, GGs, and SXNI upregulated TWEAK expression compared with the I/R group. However, the upregulation by GGs and SXNI was significantly higher than that of GFGs (**Figure 5B**). Meanwhile, Fn14 protein expression level *in situ* was detected *via* immunohistochemical staining. In MIRI mice, Fn14 staining was highly elevated in I/R groups, which was markedly reversed in I/R+GFGs and I/R+SXNI groups, and to a less content in I/R+GGs groups (**Figure 5C**). In parallel, the elevated Fn14 expression in I/R group was lowered by the treatment of GFGs, GGs, and SXNI in CIRI mice (**Figure 5D**). Taken together, these results indicate that in both heart and brain, I/R injury triggers a marked elevation of TWEAK–Fn14 expression. SXNI oppositely regulated the ligand TWEAK expression in these tissues but universally downregulated the receptor Fn14 in both heart and brain. This SXNI effect is at least in part attributable to that of GFGs or GGs in each tissue, which is consistent with the selective protection effects by GFGs or GGs in heart and brain in MIRI and CIRI mice.

### Common and Differential Targeted Genes of Ginkgo Flavonol Glycosides and Ginkgolides in MICI and CIRI

**Figure 6:**
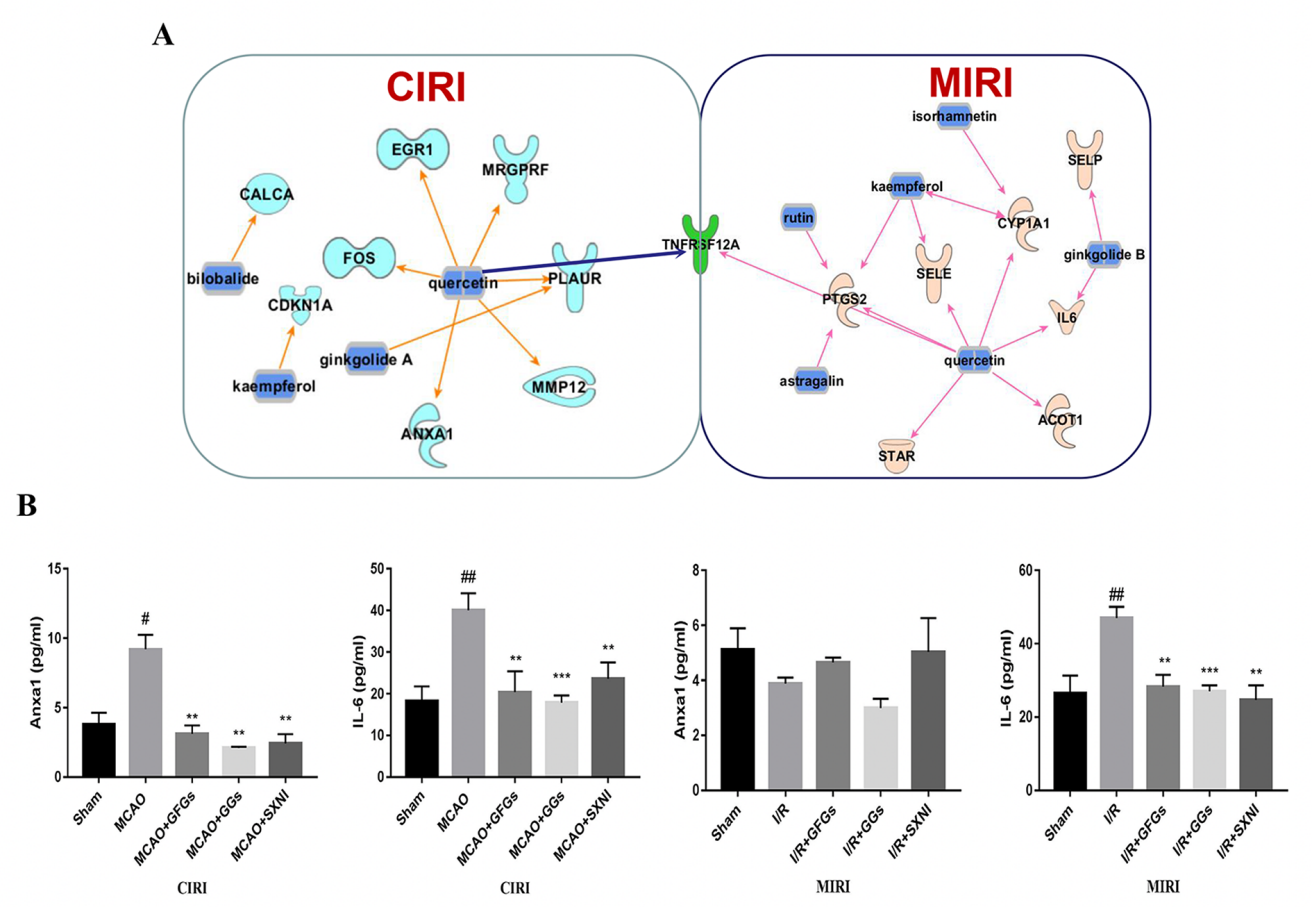
GFGs and GGs regulated common and different genes in MIRI and CIRI mice. Previously identified differentially expressed genes, databases of GFGs and GGs of SXNI and their corresponding targets were used to reveal the interaction network by Ingenuity^®^ Pathway Analysis (IPA). **(A)** The relationship between GFGs and GGs of SXNI (in blue) and their crucially targeted genes in CIRI (in light blue) and MIRI (in light purple), as well as the shared target gene (in green). **(B)** Effects of GFGs and GGs on the expression of interleukin 6 and annexin A1 in mice subjected to MIRI or CIRI (*n* = 3). Values were expressed as mean ± SD. ^#^ *P* < 0.05, ^##^ *P* < 0.01 vs. sham group, **P < 0.01, vs. model group.

Based on previous SXNI transcriptome sequencing data (Lyu et al., 2018), the targeted genes for the GFGs and GGs were identified using IPA. As depicted in **Figure 6A**, bilobalide, kaempferol, ginkgolide A, and quercetin in SXNI protected the brain from I/R injury in CIRI mice *via* regulating *Calca, Fos, Cdkn1a, Egr1, Mrgprf, Anxa1, Mmp12*, and *Plaur*. Thereinto, bilobalide and ginkgolide A belong to GGs. In addition to the regulation of *Selp* by ginkgolide B for relieving I/R injury in MIRI mice, the expression of *Ptgs2, Sele, Cyp1a1, IL6, Star, Acot1*, and *Selp* was affected by GFGs, which include kaempferol, isorhamnetin, astragatin, quercetin, and rutin. Remarkably, *Tnfrsf12a* (ENSMUSG00000023905) was detected as an overlapping gene between the MIRI and CIRI (**Figure 6A**) and was directly regulated by quercetin. Therefore, GFGs and GGs may alleviate I/R injury of heart and brain by differentially affecting the expression of Tnfrsf12a in an organ-specific manner in mice. In addition, we also validated and analyzed the targets of differential regulation of GFGs and GGs. The results indicated that GFGs and GGs can also regulate other differential targets that protect MIRI or CIRI. For example, annexin A1 was downregulated in CIRI without significant changes in MIRI (**Figure 6B**).

## Discussion

SXNI, consisting of mainly GFGs and GGs, has the characteristics of the synergism of multi-ingredients, multi-targets, and multi-pathways, producing a superior efficacy over a single drug (Shi et al., 2014; Yang et al., 2017; Lyu et al., 2018). There is an interesting new discovery in this study: the protective effect of GFGs on MIRI is more significant than that of GGs, while GGs are better than GFGs to reduce CIRI in CIRI mice. The cause of this phenomenon may be connected with the ability of the drug to permeate the blood–brain barrier (BBB). Previous reports showed that terpene trilactones and flavonoid aglycones are more BBB-permeable than glycosides and biflavones (Ma et al., 2014; Konczol et al., 2016). An increased drug concentration in brain may result in a higher pharmacological activity of GGs than GFGs in CIRI mice. Nonetheless, whether the effects of GFGs and GGs on CIRI are related to the BBB-permeability potential should be further verified in the future. However, in another aspect, GFGs may reduce the generation of oxygen-free radicals more than GGs in MIRI mice (Ran et al., 2014; Djouossi et al., 2015). Equally important, although GFGs tend to protect myocardial damage, GGs also play a certain auxiliary role and vice versa.

Furthermore, we also found that GFGs and GGs may relieve both MIRI and CIRI *via* regulating TWEAK–Fn14 cytokine receptor axis. Interestingly, TWEAK was significantly downregulated by GFGs in MIRI mice and significantly upregulated by GGs in CIRI mice. However, Fn14 was downregulated by GFGs and GGs in both MIRI and CIRI. It has been reported that TWEAK–Fn14 axis is involved in the pathogenesis of stroke *via* mediating neuronal apoptosis and breakdown of the BBB (Potrovita et al., 2004; Polavarapu et al., 2005; Yepes et al., 2005; Zhang et al., 2007; Haile et al., 2010). Both TWEAK and Fn14 in the ischemic tissue are upregulated in human ischemic stroke and CIRI mice (Potrovita et al., 2004; Yepes et al., 2005; Inta et al., 2008). Our results confirmed this conclusion (**Figure 5A and B**). Similarly, acute myocardial infarction elevates the level of TWEAK in serum in patients (Chorianopoulos et al., 2010). Recent studies have presented evidence that TWEAK plays a dual role in protecting brain damage, that is, a transient expression of TWEAK may be beneficial to alleviate the damage, but a sustained expression would worsen the situation (Burkly et al., 2007; Winkles, 2008; Mustonen et al., 2010). It has also been shown that TWEAK–Fn14 pathway is critical for the development of hypoxic–ischemic brain injury in immature animal in a gender-dependent matter. Specifically, Fn14 gene knockout is beneficial for females, whereas reduced Fn14 expression exacerbated brain injury in males (Kichev et al., 2018). Since our study only used male mice, it remains to be tested if the discordant expression of TWEAK and Fn14 in response to SXNI treatment is attributed to the sex difference. Finally, our network pharmacological analysis suggested that quercetin may directly act on TNFRSF12, a common target of both MIRI and CIRI, to protect against I/R injury. It is worth to further verify the role of quercetin in MIRI and CIRI and explore its interaction with TWEAK–Fn14 pathway.

In previous studies and this study, we predicted and verified that the TWEAK–Fn14 axis as a common target of SXNI for protection of both MIRI and CIRI (Lyu et al., 2018). However, due to the multi-target and multi-pathway characteristics of TCM, SXNI components other than GFGs and GGs may act on other important targets and pathways to alleviate MIRI or CIRI. The continuous exploration of the pharmacological effects of EGB may provide guidance and reference for the clinical application of SXNI and contribute to further understand the pharmacological mechanisms of SXNI.

## Other Limitation and Future Directions

Our current study investigated the heart and brain locally in MIRI and CIRI models separately. However, we have shown recently that CIRI could impact the heart in a model of brain–heart syndrome and another compound Chinese medicine, Danhong injection, effectively protected the heart in this model *via* local and remoteβ-AR signaling (Orgah et al., 2018). Whether SXNI and its GFG/GG components could exert a similar effect remains to be tested.

In this study, we focused on TWEAK–Fn14 axis, which was revealed as the key commonly regulated signaling pathway by SXNI in MIRI and CIRI (Lyu et al., 2018). However, our transcriptome analysis also identified differentially expressed genes by SXNI in heart and brain in an ischemia setting. For example, CYP1A1 and MMP12 may also contribute in the protective effects of GFGs and GGs in MIRI and CIRI (Lv et al., 2008; Ganesan et al., 2010; Mahdessian et al., 2017; Wilsher et al., 2017). We will further explore the roles of GFGs and GGs in regulating these differentially regulated genes and their contribution to the organ-specific protection effects in the future.

## Conclusion

We reveal for the first time the different advantages of GFGs and GGs of SXNI in protecting MIRI and CIRI, where GFGs tend to protect myocardial damage while GGs tend to relieve brain injury. Moreover, GFGs may mitigate the I/R injury of the heart in MIRI mice *via* the downregulated TWEAK–Fn14 axis, whereas GGs may upregulate the signaling pathway to protect the brain from I/R injury in CIRI mice. Overall, the study further elaborates on the material basis and mechanism of SXNI in the treatment of ischemic stroke and coronary heart disease.

## Data Availability Statement

All datasets generated for this study are included in the manuscript and the supplementary files.

## Ethics Statement

This study was performed following the recommendations in the Guidance for the Care and Use of Laboratory Animals issued by the Ministry of Science and Technology of China (permit number: TCM-LAEC2014004).The experimental procedures were based on the Directive 2010/ 63 / EU adopted by the European Union (EU), and all animals were administrated in accordance with the guidelines of Tianjin University of TCM Animal Research Committee (TCM-LAEC2014004).

## Author Contributions

YZ contributed to the conception and design of the study. ML, JN, and LL carried out the myocardial ischemia—perfusion correlative experiments provided in **Figures 1**–**3**. GX and YW carried out the cerebral ischemia—perfusion assay depicted in **Figure 4**. GX, SH, and XL participated in the relevant experiments in **Figure 5**. GX and ML performed the IPA analysis prepared in **Figure 6**. GF, XW, JH, and XG helped with the design of the study and explanation of results. YZ, GX, and ML wrote the manuscript. All authors reviewed and approved the manuscript.

## Funding

This study was supported by the grants from National Science Foundation of China (81873037), Major National Science and Technology Projects (2018YFC1704500) and China Postdoctoral Science Foundation Grant (2019M650989).

## Conflict of Interest Statement

The authors declare that the research was conducted in the absence of any commercial or financial relationships that could be construed as a potential conflict of interest.
